# Development of a ground-based sensorimotor disorientation analog to replicate astronaut postflight experience

**DOI:** 10.3389/fphys.2024.1369788

**Published:** 2024-04-18

**Authors:** Sarah C. Moudy, Brian T. Peters, Torin K. Clark, Michael C. Schubert, Scott J. Wood

**Affiliations:** ^1^ Aegis Aerospace, Houston, TX, United States; ^2^ KBR, Houston, TX, United States; ^3^ Bioastronautics Laboratory, Smead Aerospace Engineering Sciences Department, University of Colorado Boulder, Boulder, CO, United States; ^4^ Laboratory of Vestibular NeuroAdaptation, Department of Otolaryngology Head and Neck Surgery, Johns Hopkins University School of Medicine, Baltimore, MD, United States; ^5^ NASA Johnson Space Center, Houston, TX, United States

**Keywords:** spaceflight analog, sensorimotor system, functional performance, disorientation, vestibular system

## Abstract

The perceptual and motor coordination problems experienced following return from spaceflight reflect the sensory adaptation to altered gravity. The purpose of this study was to develop a ground-based analog that replicates similar sensorimotor impairment using a standard measures test battery and subjective feedback from experienced crewmembers. This Sensorimotor Disorientation Analog (SDA) included varying levels of sensorimotor disorientation through combined vestibular, visual, and proprioceptive disruptions. The SDA was evaluated on five previously flown astronauts to compare with their postflight experience and functional motor performance immediately (Return (R)+0 days) and +24 h (R+1) after landing. The SDA consisted of galvanic vestibular stimulation (GVS), visual disruption goggles, and a weighted suit to alter proprioceptive feedback and replicate perceived heaviness postflight. Astronauts reported that GVS alone replicated ∼50–90% of their postflight performance with the weighted suit fine-tuning the experience to replicate an additional 10%–40% of their experience. Astronauts did not report feeling that the disruption goggles represented either the visual disruptions or illusory sensations that they experienced, nor did they impact motor performance in postflight tasks similarly. Based on these results, we recommend an SDA including the GVS and the weighted suit. These results provide a more realistic and portable SDA framework to provide transient spaceflight-relevant sensorimotor disruptions for use in countermeasure testing and as a pre-flight training tool.

## 1 Introduction

Upon return to Earth’s gravity following prolonged microgravity exposure, astronauts experience re-entry motion sickness (Reschke et al., 2017), perceptual illusions (Harm et al., 2015), and alterations to functional performance including postural stability (Wood et al., 2015) and locomotion (Mulavara et al., 2018; Clément et al., 2022). Sensorimotor disruptions following long-duration stays on the International Space Station (ISS) have had prominent effects on functional performance immediately upon landing (Reschke et al., 2020) and +24 h after landing (Miller et al., 2018; Mulavara et al., 2018). The sensorimotor system includes the vestibular, proprioceptive, and visual systems that are critical for postural stability and gait. Not surprisingly, the postflight effects include visual orientation illusions (Oman, 2003), vestibular-mediated gain changes (Reschke et al., 2018) and changes in proprioception leading to perceived heaviness of limbs (Ross, 1998).

Simulating the postflight sensorimotor disruptions after long duration missions is exceedingly challenging on Earth. Spaceflight ground analogs such as bed rest and dry/wet immersion simulate the response to microgravity for various physiological systems (Pandiarajan and Hargens, 2020). Another spaceflight analog, centrifugation, simulates artificial gravity and can elicit vestibular adaptive changes similar to G-transitional effects following spaceflight (Bles et al., 1997; Nooij and Bos, 2007; Groen et al., 2008). Each of these spaceflight analogs provide the ability to capture large experimental datasets to test spaceflight countermeasures. However, these analogs are costly, require large facilities and significant time and effort, and most do not necessarily capture the relevant sensorimotor mechanisms. One spaceflight analog specific to sensorimotor mechanisms was developed by Dixon and Clark (2020) using a 12-h “wheelchair head immobilization paradigm” that did elicit illusory sensations and significant performance decrements in tasks sensitive to vestibular function such as tandem walk with eyes closed. However, this analog still requires significant time and effort to implement and produced limited proprioceptive and visual disruptions. A portable simple alternative that can mimic postflight sensorimotor disorientation could aid in defining sensorimotor performance thresholds and allow for faster countermeasure viability testing. Disorientation of the sensorimotor system in 1G through the same mechanisms as exposure to microgravity is difficult to replicate, however, we can replicate the motor output that is seen postflight by altering the vestibular, proprioceptive, and visual systems concurrently.

Previous studies have utilized galvanic vestibular stimulation (GVS) as a means to disrupt vestibular input and mimic astronaut postflight performance. A pseudorandom GVS profile with peaks up to 5 mA was found to significantly degrade postural stability during a computerized dynamic posturography task with eyes open and eyes closed (MacDougall et al., 2006) and performance in a locomotor obstacle course task (Moore et al., 2006). These studies found performance decrements with GVS were similar to those observed in short duration mission astronauts immediately postflight. GVS is also a portable system that can be worn while ambulating and is temporary with quick dissipation of the disruption. Thus, GVS can be a useful tool in replicating post-spaceflight task performance.

Proprioceptive functions are altered with exposure to microgravity (Macaulay et al., 2021). Vibration-induced limb position caused a greater extension perception during 1.8G parabolic flights relative to 1G (Lackner and DiZio, 1992). Lackner and DiZio (1992) proposed that these proprioceptive illusions were due to a central reinterpretation of muscle spindle stretch activity relative to gravity cues. It is possible that applying increased body-loading in 1G could elicit perceived alterations in limb position sensing. Applied loads could also mimic the subjective heaviness felt postflight by astronauts and decrements associated with reduced muscular fitness and fatigue that impact motor output and limb position sensing. Ryder et al. (2013) developed an analog to simulate spaceflight decrements in muscular fitness by applying loads upwards of 120% body weight across all body segments finding reductions in task performance as loads increased. This study found task performance was comparable to that of six astronauts after short duration shuttle missions. Distally applied loads at the ankle have also been shown to increase overall metabolic rate (Browning et al., 2007) where muscle fatigue can influence limb position sensing (Walsh et al., 2004). At the wrist, distally applied loads can increase limb position sensing error when the arm is swung (Shibata et al., 2012) and when in static unsupported condition (Winter et al., 2005). Taken together, applied body loading at the chest and distally at the wrists and ankles could elicit similar motor performance decrements as returning astronauts experience postflight.

Visual disruptions are experienced postflight including illusory sensations and reduced dynamic visual acuity (Bloomberg and Mulavara, 2003). Illusory sensations are thought to occur due to postflight readaptation of the otolith organs due to microgravity exposure where head tilt cues are reinterpreted as linear translation. This readaptation results in perceived translation of the environment or body when tilting the head after return from spaceflight which can impact postural and dynamic stability (Merfeld, 2003; Harm et al., 2015; Reschke et al., 2017). Dynamic visual acuity decreases postflight (Peters et al., 2011) and is postulated to occur due to changes in gaze stabilization mechanisms where compensatory eye movements, mediated by the vestibular system, are altered (Bloomberg and Mulavara, 2003). It was also proposed that lower-limb kinematics were altered to compensate for the changes in gaze stabilization. This visual-motor relationship, independent of cause-effect, suggests that visual distortion type challenges (e.g., Welch et al., 2009) could impact motor performance and that this could aid in replicating postflight performance.

The purpose of this exploratory study was to validate a novel post-spaceflight Sensorimotor Disorientation Analog (SDA) for replicating the postflight subjective experience in previously flown astronauts. Each of these crewmembers had participated in a sensorimotor test battery following long-duration spaceflight occurring immediately after landing (Return (R)+0 days) and 1-day after landing (R+1) (Clément et al., 2022). Therefore, both subjective feedback and comparison of performance on this test battery served as the criteria to validate the SDA.

## 2 Methods

### 2.1 Participants

The test procedures were approved through the NASA Institutional Review Board and in accordance with the Declaration of Helsinki. Five United States Orbital Segment (USOS) Astronauts (4 female, 1 male; Age: 45 ± 8 years (Mean ± SD)) who had previously flown on the ISS (average mission duration: 249 days) provided written informed consent to participate in this study. All astronauts had previously participated in sensorimotor field testing (Clément et al., 2022) immediately post-landing (1.7 ± 0.8 h) and 1-day after landing (26.9 ± 5.2 h) on their most recent return. The average time since their most recent return was 377 days (range: 95–904 days). The astronauts who participated in this study were selected based on their availability with the preference of those who had more recently returned from spaceflight in order to aid in recall. The tasks performed for this study were the same tasks completed during field testing in order to aid in recall of their experience and performance at the R+0 and R+1 time points. One astronaut did not perform field testing on R+0 but was able to perform at R+1. Feedback from this astronaut was only gathered for the R+1 time point.

### 2.2 Sensorimotor disorientation analog

The SDA consisted of three elements: GVS, visual disorientation prism goggles, and a weighted suit. Two levels of disorientation were defined as low, attempting to replicate R+1, and high, attempting to replicate R+0. The initial investigator defined starting levels for each element of the SDA are included in Table 1.

**TABLE 1:** Starting and final levels of disorientation for each element of the sensorimotor disorientation analog at each time point.

Level	GVS (peak mA) (S)	Weighted suit (%BW)	Visual disruption (BAC)
Investigator Defined Initial Starting Levels of Disorientation			
Low (i.e., aiming to replicate R+1)	2.0	20	.07–.10+
High (i.e., aiming to replicate R+0)	3.0	40	.12–.15+
Final Levels of Disorientation from Crew Feedback			
Low (i.e., aiming to replicate R+1)	2.0	15	None
High (i.e., aiming to replicate R+0)	3.0	30	None

**FIGURE 1:**
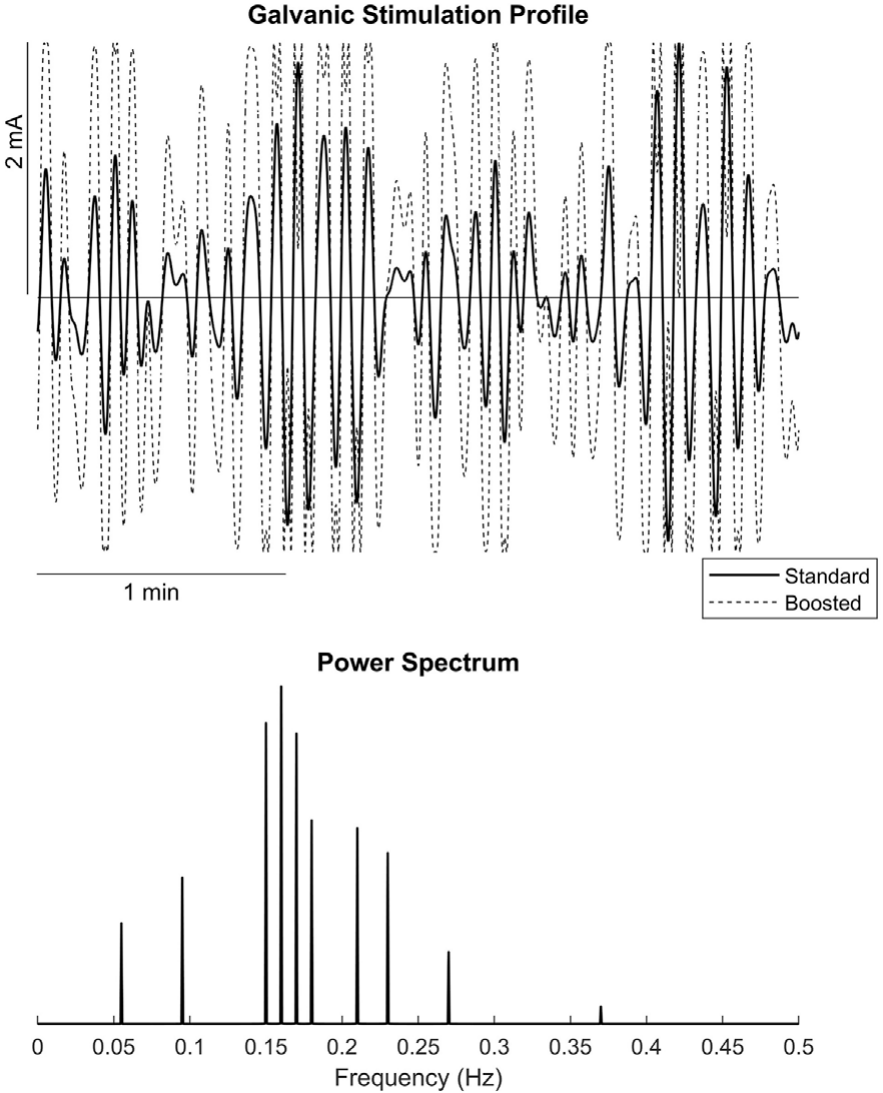
**(A)** example Standard and Boosted galvanic vestibular stimulation profiles, **(B)** power spectrum of the stimulation profiles.

S = standard profile; BW = bodyweight; BAC = blood alcohol content,

A custom GVS generator was used to deliver a bilateral bipolar stimulus. The current was delivered via two 3” diameter circular electrodes placed over the mastoid processes. An electrode pad with a layer of electrode gel was placed between the skin and the electrode. The electrodes were secured to the head via elastic straps that did not restrict head movement. The stimulus was generated from accelerometer data captured during capsule wave motions to create a random sum-of-sines profile with frequencies between 0 and 0.3 Hz (Wood, 2002; Clement and Wood, 2014). Three standard (S) profiles were generated with peak amplitudes reaching 1 mA, 2 mA, and 3 mA. Additionally, three boosted (B) profiles were generated that multiplied the standard signal to the power of 1.2 while maintaining the peak amplitude thresholds (Figure 1). Each profile contained ten, 3-min portions of non-repeating signal, for a total of 30 min, however, the GVS was only active when performing the field tasks. The six profiles were sorted by increasing levels of disorientation as defined by average peak amplitudes of the signal as follows: 1 mA S, 2 mA S, 1 mA B, 3 mA S, 2 mA B, 3  mA B.

Fatal Vision Alcohol Impairment Goggles (Innocorp, ltd., Verona, WI) were used to alter visual input. The goggles had varying levels of visual disorientation based on estimated ranges of blood alcohol content (BAC) including: 0.07–0.10+, 0.12–0.15+, and 0.17–0.20+. Prior to testing the visual disruption goggles, astronauts were told that the goggles would not replicate the actual visual disruptions or illusory sensations they experienced, if any, rather that these goggles were meant to reduce visual dependency such that task performance was similar to how postflight visual disruptions or illusory sensations could have impacted their postflight performance.

The weighted suit was comprised of the custom-made weighted vest (Ryder et al., 2013) and commercial off-the-shelf ankle and wrist straps. The vest portion only of the Ryder et al. (2013) custom-made suit was utilized for initial set-up, however, the hips and upper and lower arm and leg pieces were available to be included in testing dependent on astronaut feedback. Weights were distributed anthropometrically and symmetrically based on the relative percent weight of each body segment with respect to the overall body weight as follows: 7.3% for each wrist, 15.9% of each ankle, and 26.8% for the chest and back individually. The levels of disruption can be incrementally increased or decreased by overall percent bodyweight, with initial investigator defined starting levels noted in Table 1 for all elements.

### 2.3 Data collection procedure

Upon arrival, the astronauts were shown videos of their performance during field testing at both R+0 and R+1 to enable recall of their experience. Astronauts then performed the same field test tasks with no SDA to provide a baseline of performance. These tasks included a sit-to-stand with walk-and-turn and tandem walk. The sit-to-stand with walk-and-turn included standing from a chair, walking to and around a cone placed 400 cm away while navigating an 30 cm tall obstacle placed 130 cm away from the chair. The tandem walk was performed with both eyes open, and eyes closed including 10–12 heel-to-toe steps with arms crossed. These tasks are described in full in Clément et al. (2022).

**FIGURE 2:**
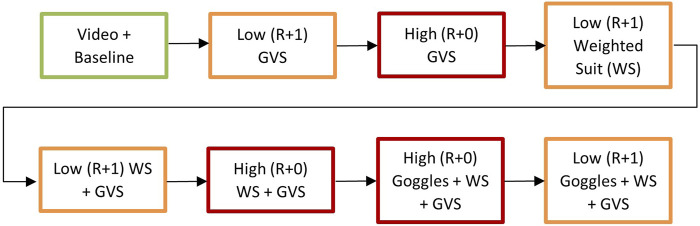
Testing procedure for each element of the Sensorimotor Disorientation Analog (SDA) with the low level (attempting to replicate R+1) outlined in orange and the high level (attempting to replicate R+0) outlined in red. Green outline represent the pre-SDA procedures. GVS = galvanic vestibular stimulation, WS = weighted suit.

Iterative testing of the SDA was performed such that the elements (GVS, vision goggles, weighted suit) were examined separately and combined as depicted in Figure 2. This approach helped determine if a singular or multiple elements of the SDA were needed to sufficiently replicate the postflight experience and performance. To note, the weighted suit alone for the high level was not performed with the tasks due to time constraints, however, subjective feedback was still captured. Throughout testing, video cameras were used to capture verbal feedback and task performance. After each block and performance of field test tasks, astronauts were asked the following questions:

• “What time point do you believe your performance and/or experience with this SDA level best reflects?”
• When applicable, “Do you believe the combination of [elements] is the same, worse, or better than with the [element] alone?”

Specific questions for each element were also asked including:

• “Is the weight and distribution of the weight similar to your postflight heaviness?”
• “Does the weight impact your ability to perform the tasks similar to postflight?”
• “Did you experience any asymmetrical heaviness postflight?”
• “Do the vision goggles impact your performance similar to your postflight performance?”

Motor performance changes were quantified via the tandem walk task. No data was gathered from the sit-to-stand with walk-and-turn task that would allow for comparison to postflight performance, rather the task was used solely to enable recall of their experience. Scoring was performed on the tandem walks when the astronaut was wearing the final preferred SDA as determined by their experience for both low and high levels. Two independent scorers examined the videos to determine percent correct steps. As in Clément et al. (2022), an incorrect step was defined as any of the following: 1) a cross-over step; 2) the stepping foot touches the ground more than once per step; 3) a wide swing of the stepping foot typically accompanied by a lateral trunk bend; 4) a step duration greater than 3 s; or 5) a heel-toe gap larger than 10 cm at the completion of the step. The average value of percent correct steps across scorers was used for each trial.

## 3 Results

In summary, the final SDA based on astronaut feedback included the GVS at the proposed starting levels and the weighted suit which was reduced to 15% and 30% body weight for the low (R+1) and high (R+0) levels respectively (Table 1). These levels were decided based on the majority consensus (GVS) or average of preferred level (weighted suit). The visual disruption goggles were removed from SDA.

These conclusions were based upon the following feedback from astronauts. All five astronauts believed the 2  mA S profile best reflected their overall experience and performance at the R+1 time point. For the R+0 time point, three of four astronauts chose the 3  mA S profile and one astronaut selected the “boosted” 2 mA profile. Four of the five astronauts (all female) subjectively reported that GVS alone replicated ∼80–90% of their postflight experience and performance with one astronaut (male) stating only 50% replicative. Two astronauts stated the GVS level for both time points was task specific such that complex tasks sensitive to vestibular function, such as tandem walk eyes closed, were more affected by GVS.

For the weighted suit element of the SDA, the final bodyweight percentage ranged from 25%–40% for the high level (R+0) and 10%–20% for the low level (R+1). The astronaut who concluded testing with 40% bodyweight for the high level (R+0) stated this was too high, however, due to time constraints this astronaut was unable to test 30% bodyweight. Overall, all the astronauts believed the weighted suit alone replicated between 5%–40% of their postflight experience and should be used alongside the GVS. All astronauts stated they did not experience asymmetrical heaviness, therefore, only a uniform application of weight was used. Three of five astronauts stated the ankle weights were useful in replicating postflight proprioceptive disruption as it reduced their ability to determine foot placement during tandem walk and disrupted the standard swing phase mechanics during the sit-to-stand with walk-and-turn. The vest aided in overall subjective heaviness; however, it was noted by the astronauts that the vest partially aided in stability during upright standing and walking tasks as the weight is around the center of mass. A few additional suggestions on weight distribution were to add light head weights to further influence head mechanics (n = 1) and distribute weight across upper and lower arms (n = 1).

Two astronauts did experience illusory sensations postflight (non-specific) and all five astronauts stated they used vision to compensate for vestibular disruptions. Four of the five astronauts did not believe the visual disruption goggles represented either the illusory sensations or other visual disruptions that they experienced postflight. Specifically, these 4 astronauts reported that the lowest BAC level of the goggles was too disruptive. Two of the four astronauts did not perform the tasks with the goggles as they either did not want to proceed after donning the goggles and standing or they were on a time constraint and wanted to focus on the other aspects of the SDA they deemed more replicative. The other two astronauts did not believe the goggles impacted their performance in postflight tasks similarly. One astronaut did believe the goggles aided in fine-tuning the SDA to achieve an additional 5% of their postflight experience. This astronaut felt the 0.07–0.10+ BAC replicated R+1 and 0.12–0.15+ BAC replicated R+0.

Changes in motor performance while wearing the astronauts’ preferred SDA is summarized in Table 2 using percent correct steps during tandem walk. The preferred SDA for four of the five astronauts included only the GVS and weighted suit whereas the fifth astronaut also included the visual disruption goggles. In comparison to published postflight data inclusive of the astronauts in this study (Clement et al., 2022), the SDA elicited on average 10% and 6.5% less correct steps for the low level (R+1) with eyes open and closed, respectively (Table 2). Individually, each astronaut ranged from 2.4%–40% correct steps different than their respective postflight R+1 data for eyes open and 0%–22.5% correct steps different for eyes closed. Eyes closed performance for the high level (R+0) had 2.3% less correct steps when wearing the SDA compared to postflight and, individually ranged from 1.7%–17.5% corrects steps different. Conversely, performance was better with 19.8% more correct steps while wearing the SDA for eyes open at the high level (R+0), and individually ranged from 10% correct steps worse with the SDA or between 5.7%–45% corrects steps better with the SDA.

**TABLE 2:** Percent correct steps for tandem walk with eyes open and closed while wearing the Sensorimotor Disorientation Analog (SDA) at the high and low levels and postflight data retrieved from Clement et al. (2022) at the R+0 and R+1 time points.

	High Level/R+0		Low Level/R+1	
	Eyes open (%)	Eyes closed (%)	Eyes open (%)	Eyes closed (%)
SDA (R+0: n = 4, R+1: n = 5) (Mean, [Min, Max])	53.3 [18–75]	7.6 [0–19]	74.4 [56–100]	18.8 [12–30]
Clement et al. (2022) (n = 19) (Mean, [Min, Max])	33.5 [0–100]	9.9 [0–33]	84.6 [9–100]	25.3 [0–58]

Clement et al. (2022) data retrieved from supplementary file. Average of the mean data for repeat and first flight was used.

## 4 Discussion

The results of this study demonstrated that combining a GVS sum-of-sines profile with a weighted suit was able to approximately replicate, based upon subjective comparisons, astronaut immediate and +24 h postflight experience and motor performance during dynamic locomotor tasks. While no analog can fully replicate the adaptations that occur due to spaceflight, the SDA proposed in this study provides a framework for a portable spaceflight analog.

GVS alone was the best at replicating postflight experience after long duration missions. This is consistent with previous studies that utilized only GVS to successfully replicate short duration postflight postural stability and dynamic task performance (MacDougall et al., 2006; Moore et al., 2006). One astronaut noted that postflight vestibular disruptions were greater when head movements were performed including head movement relative to the body (e.g., head pitch to view obstacle on the ground) and relative to space (e.g., sit-to-stand with head locked to trunk). Moore et al. (2006) examined a head-coupled GVS profile using head yaw velocity and vertical linear acceleration to elicit proportional galvanic stimulus. These results found that the head-coupled GVS significantly disrupted performance yet produced less disruption to motor performance than a pseudorandom GVS profile similar to that used in this study. The head-coupled GVS profile utilized only yaw motion, where head pitch and roll have been reported to cause illusory sensations of exaggerated translational motion postflight (Reschke and Clément, 2018). It is also possible that the proportional relationship between head movement and galvanic stimulus was not disruptive enough to replicate astronaut’s postflight experiences. Further research is needed to determine the validity of head-coupled GVS profiles to better reflect postflight experience.

The weighted suit received overall positive feedback on replicating subjective heaviness and eliciting certain proprioceptive disruptions that are experienced following spaceflight (Ross, 1998). However, the suit was only helpful in replicating postflight experience in combination with the GVS as the visual and vestibular systems were able to compensate for the added weight. One astronaut in this study noted that the weighted suit helped to stabilize them while performing the tandem walk. Applied loads to body segments is known to impair postural limits of stability (Holbein and Chaffin, 1997), however, loading of small weights has been used therapeutically to improve postural alignment (Widener et al., 2020). It is possible that the location of the weights, specifically at the chest, may have aided in stability. Conversely, the distally applied loads at the ankle were noted by astronauts to subjectively impair task performance similar to postflight experience.

While we did not examine limb position sensing error, we did quantify motor performance during the tandem walk task as a result of the preferred combined SDA. Overall, tandem walk performance with the SDA was within 10% correct steps (∼1 step) of the average postflight performance from previous research except for eyes open at the high level (R+0) where performance was better with 19.8% more correct steps (∼2 steps) with the SDA. Postflight task performance soon after landing is highly variable, especially when able to utilize vision. This is seen in the Clément et al. (2022) data (Table 2) where the full possible range of postflight performance (0%–100% correct steps) was captured on R+0 for tandem walk eyes open. This range of performance was similarly replicated by the SDA resulting in a range of performance from 18%–75% correct steps. The final preferred SDAs across astronauts were similar, although not exact. This suggests that motor output in response to the SDA can vary by person which is consistent with postflight readaptation response.

The visual disruption goggles were utilized as a means to reduce visual dependency and, therefore, alter motor output. However, even with this pre-emptive statement, the negative feedback received about the goggles suggests that replicating the sensory experience is important. On closer examination of the one astronaut that did include the visual disruption goggles in their final preferred SDA, tandem walk scores decreased relative to without the visual disruption goggles (SDA low level (R+1): 100%–65%; high level (R+0): 92%–18% correct steps). With the goggles, SDA performance for the high level (R+0) more closely resembled their actual postflight performance. However, for the low level (R+1) their SDA performance was much worse than their actual postflight performance where performance without the goggles more accurately represented postflight. Visual dependency was calculated for all astronauts in this study from the difference in preflight tandem walk scores between eyes open and closed. The absolute differences ranged from 11%–34% correct steps with the astronaut who included the visual disruption goggles in their final preferred SDA had a 12.5% correct step difference. This suggests they are on the lower end of visual dependency compared to the others in this study. Finally, when examining individual differences in tandem walk scores between the SDA and postflight performance, eyes open had larger differences up to 45% correct steps different in comparison to eyes closed with up to 18.9% correct steps different. Taken together, this suggests that a visual disruption could improve the SDA, however, it is unclear when a visual disruption should be included. If a visual disruption is to be used, it is clear that the disruption should better replicate the postflight sensory experience such as eliciting an exaggerated movement of the surrounding environment relative to the voluntary real head movement and/or including a time delay of movement of the surrounding environment (Harm and Parker, 1993). Further research is needed to provide similar illusory sensations more accurately such as through virtual or augmented reality.

While the results from this study were compiled from a small sample of astronauts, the subjective feedback from those who relatively recently experienced these difficult to describe sensations is invaluable. One limitation of this study is that not all test conditions (e.g., visual goggles alone) were tested. It is possible that the test sequence could have influenced the subjective evaluations. Therefore, the proposed SDA is presented as a starting framework for a portable analog that requires further validation. Without prompting, three of the five astronauts suggested that the non-head-coupled GVS profile alone could be a useful preflight training tool for first time flyers. The portable SDA allows for out-of-lab field testing and provides a relatively quick reversible disorientation (Dilda et al., 2014). The SDA may be useful in future investigations on spaceflight countermeasure testing and understanding adaptation and compensatory mechanisms of the broader vestibular loss community.

## Data Availability

The original contributions presented in the study are included in the article/Supplementary material, further inquiries can be directed to the corresponding author.
